# Male external genital skin disorders: a retrospective analysis from a tertiary hospital in China

**DOI:** 10.3389/fmed.2025.1630632

**Published:** 2025-12-03

**Authors:** Yue-Tong Qian, Xiao Ma, Jing-Yi Zhang, Jia-Wei Liu, Kai Fang, Dong-Lai Ma

**Affiliations:** State Key Laboratory of Complex Severe and Rare Diseases, Department of Dermatology, Peking Union Medical College Hospital, Chinese Academy of Medical Sciences and Peking Union Medical College, National Clinical Research Center for Dermatologic and Immunologic Diseases, Beijing, China

**Keywords:** male external genitalia, skin diseases, histopathology, dermoscopy, diagnostic discrepancy

## Abstract

**Background:**

Male genital dermatoses encompass a diverse range of infectious, inflammatory, and neoplastic conditions with overlapping clinical presentations, often leading to diagnostic challenges. Misdiagnosis can result in inappropriate management and significant patient distress, particularly given the sensitive nature of the anatomical site. Although histopathology remains the diagnostic gold standard, non-invasive tools such as dermoscopy are becoming increasingly valuable for preliminary evaluation. A thorough understanding of the clinical features associated with male genital dermatoses is essential for achieving accurate diagnoses.

**Methods:**

Our retrospective study analyzed 572 histopathologically confirmed cases of lesions on the male external genitalia at Peking Union Medical College Hospital from 2015 to 2025. Data included demographics, disease duration, lesion distribution, dermoscopy results if exists, and clinical-dermatoscopic-pathological concordance.

**Results:**

The cohort with a mean age of 47.6 years, exhibited distinct age-specific patterns: adolescents (1.2%) had inflammatory/autoimmune conditions; adults (70.6%) showed human papillomavirus-associated (Bowenoid papulosis, genital warts) and inflammatory diseases (lichen sclerosus); and elderly patients (28.1%) had higher malignancy rates (extramammary Paget’s disease, squamous cell carcinoma). Overall, inflammatory diseases (33.7%) and tumors (33.4%) were the most common diagnoses. Notably, diagnostic clinical-pathological discordance was observed in 34.6% of cases, particularly for lichen planus (4.9%) and extramammary Paget’s disease (2.8%).

**Conclusion:**

This study highlights the diverse etiologies of male genital dermatoses and underscores the necessity of age-stratified evaluations. The use of dermoscopy improves diagnostic accuracy and minimizes the need for unnecessary biopsies. Conditions such as extramammary Paget’s disease, bowenoid papulosis, and lichen sclerosus require heightened awareness due to their potential for malignancy or diagnostic complexity. Integrating clinical, dermoscopic, and histopathological data is critical for effective management.

## Introduction

Male genital dermatoses encompass a wide range of conditions with overlapping clinical manifestations, including infectious, inflammatory, and neoplastic disorders, which often lead to diagnostic challenges. These dermatoses may be localized to the genital area or represent a manifestation of a more widespread dermatologic condition. Misdiagnosis or delayed diagnosis can result in inappropriate management and significant psychological distress for patients. Patients often delay seeking medical attention owing to the sensitive location of lesions, increasing risks of delayed or incorrect diagnoses and subsequent therapeutic delays. Meanwhile, patients frequently worry that genital lesions may indicate sexually transmitted infections (STIs), a concern that can lead to significant psychological distress and social stigma. This highlights the crucial need for accurate differentiation between STI-related and non-STI-related dermatoses during clinical evaluations (1). Although histopathological examination remains the gold standard for diagnosis, biopsies of the external genitalia carry risks, including potential impacts on function, appearance, psychological well-being, and overall quality of life. To better understand the clinical characteristics of these conditions, we conducted a retrospective analysis of demographic data, disease profiles, lesion morphology, and clinical-dermatocopic-pathological diagnostic discrepancies in male external genitalia biopsies performed at our center over the past decade. This study aims to enhance the recognition of these conditions among dermatologists and urologists while reducing the likelihood of misdiagnosis.

## Materials and methods

In this retrospective study, we analyzed 572 cases of histopathologically confirmed skin lesions of the male external genitalia (defined as involving the penis and/or scrotum) at Peking Union Medical College Hospital from March 2015 to March 2025. Only cases with clinical photographs, histopathological images, and comprehensive medical records were included. Data were extracted from electronic medical records, including demographics, disease duration, disease distribution, clinical presentation, symptoms, pathological diagnosis, and clinical diagnosis. All patients scheduled for skin biopsy will be screened for hepatitis B, hepatitis C, syphilis, and human immunodeficiency virus, as well as whole blood routine and coagulation tests. Additionally, for cases with available dermoscopy data, those findings were also incorporated into the analysis. All dermoscopic interpretations and histopathological evaluations were independently assessed and confirmed by two experienced dermatologists. The final histopathological results will refer to laboratory results, especially in the case of infectious diseases, for accurate diagnosis. The study was approved by the ethics committee of Peking Union Medical College Hospital (K 8437).

## Results

### Patient characteristics

The mean age of the patients was 47.64 ± 17.6 years (range: 12–95 years), with 7 cases (1.2%) occurring in adolescents aged 18 and under, 404 (70.6%) in adults (19–60 years), and 161 (28.1%) in elderly patients (>60 years,70.34 ± 17.60 years). The distribution of patients across different disease is presented in Table 1.

**TABLE 1:** Disease constitution, course and age distribution.

Disease types	Number of patients, *n*				Age, year (average ± SD)	Disease course, month (average ± SD)	Clinical misdiagnosis, *n*			
	Total	Age < 18 years	Age 19–60 years	Age > 60 years			Total (%)	Age < 18 years	Age 19–60 years	Age > 60 years
Malignant and precancerous tumors	**160**	**0**	**58**	**102**	**62.66 ± 13.20**	**34.63 ± 43.55**	**39 (24.4%)**			
Extramammary Paget’s disease	68	0	10	58	68.87 ± 8.82	50.51 ± 50.19	16 (23.5%)	0	4	12
Squamous cell carcinoma	44	0	19	25	58.97 ± 13.49	14.93 ± 14.45	4 (9%)	0	1	3
Verrucous carcinoma	16	0	13	3	52.81 ± 12.16	16.44 ± 14.09	6 (37.5%)	0	4	2
Bowen disease	7	0	6	1	54 ± 9.40	45.71 ± 51.54	3 (42.9%)	0	2	1
Basal cell carcinoma	6	0	1	5	66.5 ± 9.22	16.67 ± 15.64	3 (50%)	0	0	3
Erythema hyperplasia	17	0	8	9	57.41 ± 17.44	43.76 ± 60.09	7 (41.2%)	0	4	3
B-cell lymphoma	1	0	0	1	75	2	0	0	0	0
Kaposi sarcoma	1	0	1	0	33	3	0	0	0	0
Benign tumors	**79**	**4**	**69**	**6**	**37.37 ± 15.36**	**65.96 ± 77.10**	**29 (36.7%)**			
Melanocytic nevus	18	1	17	0	29.44 ± 8.00	74.83 ± 90.44	3 (16.7%)	0	3	0
Skin calcium deposits	12	0	12	0	34.92 ± 8.35	147 ± 112.82	1 (8.3%)	0	1	0
Seborrheic keratosis	10	0	9	1	48.6 ± 13.24	40.08 ± 45.88	8 (80%)	0	7	1
Angioceroderma	9	1	6	2	42.67 ± 22.96	51.81 ± 40.90	0	0	0	0
Genital melanosis	7	0	7	0	35.43 ± 15.67	55.71 ± 45.79	4 (57.1%)	0	4	0
Lentigo	5	0	5	0	37.8 ± 7.29	48.2 ± 48.50	5 (100%)	0	5	0
Epidermoid cyst	5	2	2	1	37.2 ± 27.07	32.4 ± 10.04	4 (80%)	1	3	0
Dermatofibroma	2	0	2	0	30.5 ± 4.95	30 ± 8.49	0	0	0	0
Soft fibromas	2	0	2	0	27.5 ± 0.71	63 ± 80.61	1 (50%)	0	1	0
Keloid	2	0	0	2	70 ± 4.24	19 ± 24.04	1 (50%)	0	0	1
Blue nevus	1	0	1	0	30	60	0	0	0	0
Epidermolytic acanthoma	1	0	1	0	49	120	1 (100%)	0	1	0
Solitary reticular histiocytoma	1	0	1	0	40	0.5	0	0	0	0
Spiroma	1	0	1	0	29	1	1 (100%)	0	1	0
Ectopic sebaceous glands	1	0	1	0	31	12	0	0	0	0
Verruciform xanthoma	1	0	1	0	55	12	0	0	0	0
Sebaceous cyst	1	0	1	0	20	24	0	0	0	0
Infectious disease	**129**	**0**	**119**	**10**	**38.66 ± 13.19**	**18.36 ± 26.09**	**55 (42.6%)**			
Bowenoid papulosis	57	0	51	6	38.18 ± 12.95	19.13 ± 24.30	24 (42.1%)	0	21	3
Gential warts	48	0	44	4	40.19 ± 13.85	23.49 ± 31.52	26 (54.2%)	0	26	0
Syphilis	12	0	12	0	34.42 ± 13.29	3.38 ± 6.69	3 (25%)	0	3	0
Herpes simplex virus	5	0	5	0	45 ± 13.36	4.69 ± 7.60	0	0	0	0
Scabies	4	0	4	0	36.5 ± 11.96	11.5 ± 16.44	1 (25%)	0	1	0
Molluscum contagiosum	3	0	3	0	31 ± 3.61	1.42 ± 1.01	1 (33.3%)	0	1	0
Inflammatory disease	**191**	**2**	**151**	**38**	**45.42 ± 15.99**	**34.90 ± 53.38**	**71 (37.2%)**			
Lichen sclerosus	57	1	50	6	41.07 ± 14.34	41.95 ± 49.33	15 (26.3%)	0	12	3
Lichen planus	54	0	48	6	43.45 ± 12.32	15.76 ± 25.72	28 (51.9%)	0	25	4
Plasma cell balanitis or Zoon’s balanitis	29	0	14	15	55.72 ± 17.89	30.28 ± 37.00	10 (34.5%)	0	7	3
Dermatitis or eczema	25	1	16	8	52.48 ± 17.97	57.094 ± 94.07	6 (24%)	0	3	3
Psoriasis	17	0	16	1	37.88 ± 10.84	40.55 ± 61.08	9 (52.9)	0	8	1
Porokeratosis	5	0	3	2	55.4 ± 24.85	79.2 ± 51.33	2 (40%)	0	1	1
Lichen nitidus	2	0	2	0	27.5 ± 3.54	12.5 ± 16.26	1 (50%)	0	1	0
Intertrigo	1	0	1	0	27	4	0	0	0	0
Pyoderma gangraenosum	1	0	1	0	52	3	0	0	0	0
Autoimmune disease and others	**13**	**1**	**8**	**4**	**47.23 ± 18.24**	**32.92 ± 48.79**	**4 (30.8%)**			
Pemphigus	3	0	1	2	62 ± 6.93	10 ± 3.46	2 (66.7%)	0	2	0
Vitiligo	3	1	2	0	23.33 ± 9.02	18 ± 10.39	1 (33.3%)	1	0	0
Drug adverse effect	3	0	1	2	63.33 ± 9.07	19.67 ± 24.54	1 (33.3%)	0	0	1
Hailey-Hailey disease	1	0	1	0	53	120	0	0	0	0
Bullous pemphigoid	1	0	1	0	36	3	0	0	0	0
Bachet disease	1	0	1	0	31	6	0	0	0	0
Reiter disease	1	0	1	0	48	156	0	0	0	0

The bold values indicate different categories of the disease types of male genital dermatoses.

### Disease constitution, duration and anatomical distribution

The study identified 47 distinct pathological diagnoses. The mean disease duration was 35.2 ± 51.9 months, with cases categorized as follows: inflammatory diseases (191 cases, 33.4%), benign and malignant tumors (239 cases, 41.8%), infectious diseases (129 cases, 22.6%), and autoimmune diseases and other conditions (13 cases, 2.3%). The ten most prevalent diagnoses were extramammary Paget’s disease (EMPD) (68 cases, 11.9%), Bowenoid papulosis (BP) (57 cases, 10.0%), lichen sclerosus (LS) (57 cases, 10.0%), lichen planus (LP) (54 cases, 9.4%), genital warts (48 cases, 8.4%), squamous cell carcinoma (SCC) (44 cases, 7.7%), balanitis (29 cases, 5.0%), dermatitis or eczema (25 cases, 4.4%), melanocytic nevi (18 cases, 3.1%), and erythema hyperplasia (17 cases, 3.0%). Demographic information, disease distribution, disease course, and age distribution are summarized in Table 1.

In the pediatric group, the mean disease duration was 38.6 ± 46.5 months. The most prevalent diagnosis was epidermoid cysts (2 cases, 28.6%), followed by vitiligo, angiokeratoma, dermatitis, LS and pigmented nevus (1 case each). Among adults, the mean disease duration was 35.5 ± 54.7 months, with infectious and inflammatory conditions predominating. The top five diagnoses in this group were BP (51 cases, 12.6%), LS (50, 12.4%), LP (47, 11.6%), genital warts (43, 10.6%), and SCC (19, 4.7%). In the elderly cohort, the mean duration was 34.5 ± 44.7 months, and there was a notable increase in the prevalence of malignancies. The top five diagnoses included EMPD (58 cases, 36.0%), SCC (25, 15.5%), balanitis (15, 9.3%), erythema hyperplasia (9, 5.6%), and dermatitis/eczema (8, 5.0%). The top five diseases across different age groups are summarized in Table 2.

**TABLE 2:** The top six diseases in different age groups.

Top rank disease	Age < 18 years (*n*)	Age 19–60 years (*n*)	Age > 60 years (*n*)
1	Epidermoid cyst (2)	Bowenoid papulosis (51)	Extramammary Paget’s disease (58)
2	Lichen sclerosus (1)	Lichen sclerosus (50)	Squamous cell carcinoma (25)
3	Melanocytic nevus (1)	Lichen planus (48)	Plasma cell balanitis or Zoon’s balanitis (15)
4	Dermatitis or eczema (1)	Gential warts (44)	Erythema hyperplasia (9)
5	Angioceroderma (1)	Squamous cell carcinoma (19)	Dermatitis or eczema (8)
6	Vitiligo (1)	Melanocytic nevus (17)	Lichen sclerosus (6)

Based on the biopsy sites of skin lesions, the male genitalia area was divided into four anatomical regions: the scrotum, penile shaft, coronal sulcus, and glans penis. The glans penis was the most frequently biopsied site (195 cases), followed by the penile shaft (178 cases), scrotum (161 cases), and coronal sulcus (38 cases). Lesions involved multiple genital regions in 61 patients, with some cases also extending to extragenital areas (e.g., perianal region, perineum, and axillae). The distribution of these disease is presented in Table 3.

**TABLE 3:** Distribution of different male external genital diseases.

Disease types	Scrotum (*n* = 161)	Coronal sulcus (*n* = 38)	Glans penis (*n* = 195)	Penile shaft (*n* = 178)
**Malignant and precancerous tumors**				
Extramammary Paget’s disease	60	1	0	7
Squamous cell carcinoma	4	1	22	17
Verrucous carcinoma	0	2	10	4
Bowen disease	2	0	1	4
Basal cell carcinoma	3	0	1	2
Erythema hyperplasia	0	0	16	1
B-cell lymphoma	0	**0**	**0**	**1**
Kaposi sarcoma	0	0	1	0
**Benign tumors**				
Melanocytic nevus	2	1	10	5
Skin calcium deposits	12	0	0	0
Seborrheic keratosis	5	0	0	5
Angioceroderma	7	0	2	0
Genital melanosis	0	1	3	3
Lentigo	2	0	1	2
Epidermoid cyst	3	0	0	2
Dermatofibroma	1	0	0	1
Soft fibromas	2	0	0	0
Keloid	0	1	0	1
Blue nevus	0	0	0	1
Epidermolytic acanthoma	1	0	0	0
Solitary reticular histiocytoma	1	0	0	0
Spiroma	0	0	0	1
Ectopic sebaceous glands	0	0	0	1
Verruciform xanthoma	1	0	0	0
Sebaceous cyst	1	0	0	0
**Infectious disease**				
Bowenoid papulosis	8	9	3	37
Gential warts	8	5	4	31
Syphilis	5	3	3	1
Herpes simplex virus	0	0	1	4
Scabies	3	0	0	1
Molluscum contagiosum	0	1	0	2
**Inflammatory disease**				
Lichen sclerosus	0	6	37	14
Lichen planus	0	6	34	14
Plasma cell balanitis or Zoon’s balanitis	0	0	21	8
Dermatitis or eczema	22	0	0	3
Psoriasis	0	0	16	1
Porokeratosis	4	0	0	1
Lichen nitidus	1	0	1	0
Intertrigo	1	0	0	0
Pyoderma gangraenosum	0	0	1	0
**Autoimmune disease and others**				
Pemphigus	0	0	2	1
Vitiligo	1	0	1	1
Drug adverse effect	0	0	2	1
Hailey-Hailey disease	1	**0**	**0**	**0**
Bullous pemphigoid	0	0	**1**	0
Bachet disease	0	1	0	0
Reiter disease	0	0	1	0

The bold values indicate different categories of the disease types of male genital dermatoses.

### Clinical presentations

Dermatoses affecting the male external genitalia present a wide range of clinical manifestations. In our study, the manifestations of the skin diseases were consistent with previous findings. EMPD was characterized by persistent erythematous plaques, often accompanied by exudation, erosion, or ulceration. LS typically presented with localized sclerosis and epidermal atrophy, with severe cases progressing to frenular contracture and urethral stricture, resulting in functional impairment. Malignant neoplasms, including basal cell carcinoma (BCC), SCC, and verrucous carcinoma, typically manifested as nodules or indurated masses, sometimes with chronic non-healing ulcers.

**FIGURE 1:**
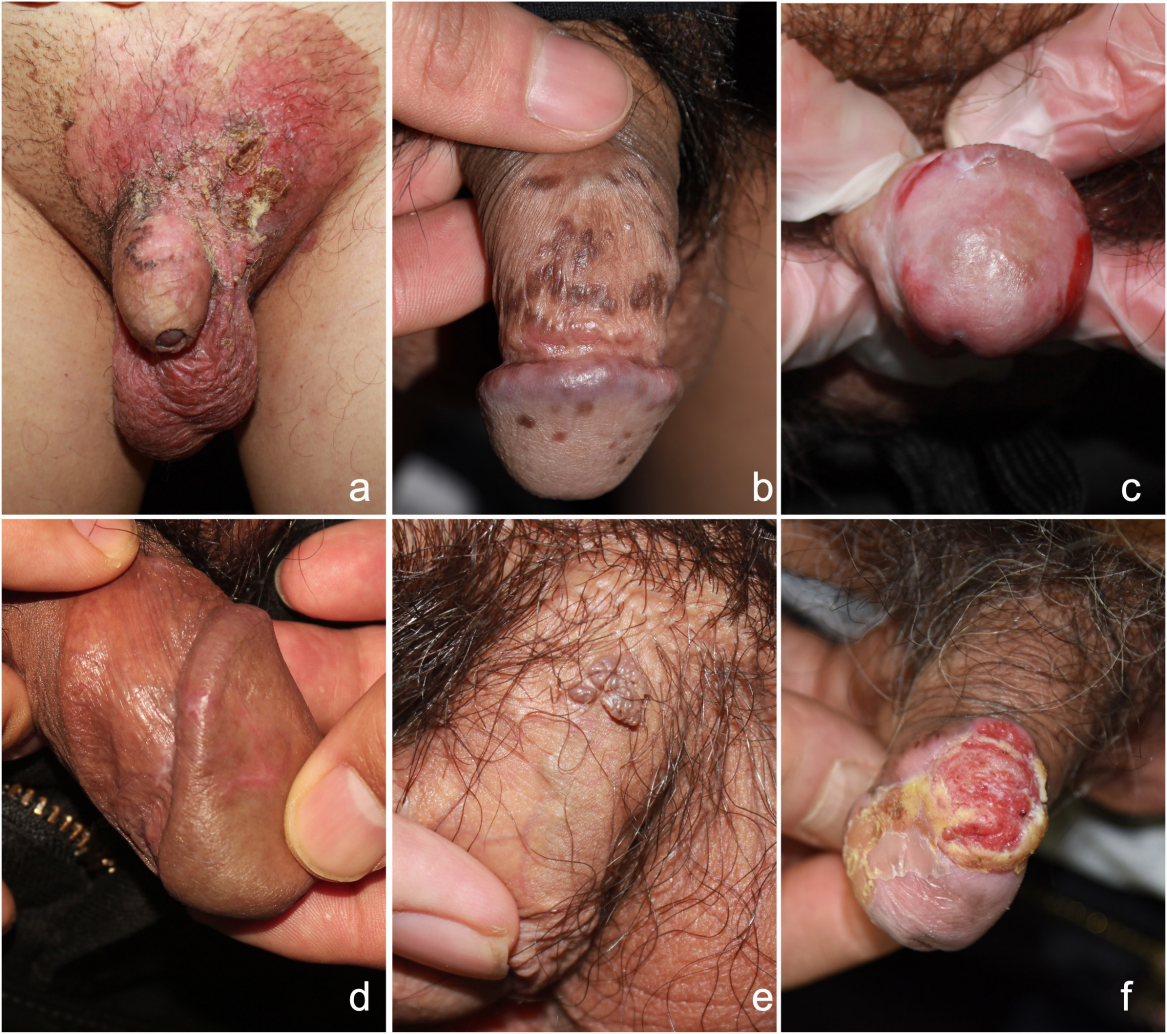
Clinical features of common male external genital skin diseases. **(a)** Extramammary Paget disease: Ill-defined erythematous plaque with exudation, erosion and scales involved the scrotum and penile shaft. **(b)** Bowenoid papulosis: Multiple pigmented maculopapular lesions with various sizes on the glans penis and penile shaft. **(c)** Lichen sclerosus: Porcelain-white atrophic plaques with epidermal fragility, and erosions on the glans penis. **(d)** Lichen planus: Violaceous polygonal papules with white reticular striae along the coronal sulcus and glans. **(e)** Genital warts: Multiple skin-colored to hyperpigmented cauliflower-like papulonodules distributed on the penile shaft. **(f)** Squamous cell carcinoma: An erythematous verrucous mass exhibiting central erosive ulceration surrounded by peripheral hyperkeratosis with serous crusting and collarette scaling on glans penis.

Erosion and ulcers can also be seen in infectious skin diseases such as syphilis and herpes simplex, as well as in autoimmune bullous disease. Soft fibromas typically present as pedunculated skin tags. Papular lesions with variable coloration, size, and surface texture were observed in conditions such as BP, warts, lichen nitidus, and sebaceous hyperplasia. Scaling plaques were prominent in psoriasis and lichen planus, while erythroplasia of Queyrat and balanitis primarily presented as erythematous patches. Pruritus was a hallmark of inflammatory conditions (e.g., dermatitis, eczema, lichen simplex chronicus), whereas pain was strongly associated with ulcerative lesions. A summary of these clinical manifestations is summarized in Figure 1 and Table 4. The supplementary figures depict the typical dermoscopic (Supplementary Figure 1) and histopathological (Supplementary Figure 2) features of common male external genital skin diseases.

**TABLE 4:** Clinical features of the male external genital diseases.

Disease types malignant and precancerous tumors	Macule/patch	Papule/plaque	Nodule/mass	Atrophy/sclerosis	Exudation/erosion/ulcer	Scale
Extramammary Paget’s disease	62	2	5	0	15	2
Squamous cell carcinoma	9	7	17	0	18	1
Verrucous carcinoma	2	5	10	0	1	0
Bowen disease	5	1	1	0	0	1
Basal cell carcinoma	3	1	2	0	1	0
Erythema hyperplasia	14	1	1	0	3	1
B-cell lymphoma	0	0	1	0	0	0
Kaposi sarcoma	1	0	0	0	0	0
**Benign tumors**						
Melanocytic nevus	17	1	0	0	0	0
Skin calcium deposits	0	1	11	0	0	0
Seborrheic keratosis	4	5	1	0	0	0
Angioceroderma	1	8	0	0	0	1
Genital melanosis	6	1	0	0	0	0
Lentigo	3	2	0	0	0	0
Epidermoid cyst	1	0	4	0	0	0
Dermatofibroma	0	1	1	0	0	0
Soft fibromas	0	0	2	0	0	0
Keloid	1	0	1	0	0	0
Blue nevus	0	0	1	0	0	0
Epidermolytic acanthoma	0	1	0	0	0	0
Solitary reticular histiocytoma	0	1	0	0	0	0
Spiroma	0	1	0	0	0	0
Ectopic sebaceous glands	0	1	0	0	0	0
Verruciform xanthoma	0	0	1	0	0	0
Sebaceous cyst	0	0	1	0	0	0
**Infectious disease**						
Bowenoid papulosis	28	26	3	0	0	0
Gential warts	4	42	2	0	0	0
Syphilis	3	1	0	0	7	1
Herpes simplex virus	2	1	0	0	2	0
Scabies	0	2	2	0	0	0
Molluscum contagiosum	0	3	0	0	0	0
**Inflammatory disease**						
Lichen sclerosus	28	4	2	31	4	1
Lichen planus	39	12	0	0	7	5
Plasma cell balanitis or Zoon’s balanitis	21	2	0	0	10	3
Dermatitis or eczema	20	3	3	0	5	1
Psoriasis	12	4	0	0	0	4
Porokeratosis	2	2	0	0	0	0
Lichen nitidus	0	2	0	0	0	0
Intertrigo	1	0	0	0	0	0
Pyoderma gangraenosum	0	0	0	0	1	0
**Autoimmune disease and others**						
Pemphigus	3	0	0	0	3	0
Vitiligo	3	0	0	0	0	0
Drug adverse effect	2	**1**	0	0	0	0
Hailey-Hailey disease	1	0	0	0	1	0
Bullous pemphigoid	0	0	0	0	1	0
Bachet disease	0	0	1	0	0	0
Reiter disease	1	1	0	0	0	0

The bold values indicate different categories of the disease types of male genital dermatoses.

### Inconsistency between the clinical and pathological diagnosis

In our retrospective study, 198 cases (34.6%) exhibited discordance between the initial clinical diagnoses and the final pathological diagnoses. Our study revealed variable discordance rates across disease categories, with the highest rates observed in infectious diseases (42.6%), followed by inflammatory conditions (37.2%), benign tumors (36.7%), autoimmune and other disorders (30.8%), and malignant or premalignant lesions (24.4%). The discordance rate is presented in detailed in Table 1.

### Inconsistency between the clinical and dermoscopy diagnosis

Using the patient names and medical record numbers, we systematically cross-referenced our institutional dermoscopic database with histopathological records, identifying 73 cases (16.9% of 433 histopathologically evaluated patients) with complete clinical photographs and dermoscopic records. Through analysis of characteristic dermoscopic patterns, we achieved definitive diagnoses of seborrheic keratosis, LS, psoriasis, angiokeratoma, melanocytic nevus, porokeratosis, balanitis, genital warts, LP, BP, and malignant conditions. Histopathological confirmation was obtained in 42 cases (57.5% diagnostic concordance rate). Eighteen cases (24.7%) exhibited non-specific dermoscopic patterns necessitating comprehensive differential diagnosis, encompassing inflammatory conditions (balanitis, LP, LS, dermatitis), infectious diseases (syphilis, BP), and neoplastic lesions including EMPD and erythroplasia of Queyrat. The dermoscopic diagnosis of 13 cases was different from the final pathological diagnosis, including the confusion between genital warts and BP, and the confusion between LS and LP.

## Discussion

Our retrospective study evaluated 572 histopathologically confirmed cases from Peking Union Medical College Hospital, encompassing a comprehensive assessment of demographic data, disease duration, clinical manifestations, and diagnostic concordance between initial clinical impressions and definitive histological findings. STIs, including syphilis, herpes simplex virus (HSV) infection, and gonorrhea, represent the most prevalent conditions affecting the male external genitalia. In contrast, non-STI dermatoses of the male external genitalia encompass a wide spectrum of disease with varied etiologies. There are only a few comprehensive studies on the pattern of nonvenereal dermatoses in males (2, 3). To the best of our knowledge, our study is the largest single-center study of male external genitalia skin diseases in China to date. Our cohort demonstrated remarkable etiological diversity, incorporating infectious pathogens, immune-mediated conditions, chronic inflammatory disorders, and metabolic deposition diseases, thereby providing a representative cross-section of genital dermatopathology in male patients.

In our study, we observed significant differences in the disease spectrum among various age groups. Adolescents predominantly affected by inflammatory and autoimmune conditions, while sexually active adults showed a higher prevalence of human papillomavirus (HPV)-associated diseases. Notably, elderly patients had a significantly elevated risk of developing cutaneous tumors, emphasizing age as a critical oncogenic risk factor. These findings highlight the importance of age-stratified diagnostic approaches. In particular, for elderly populations, proactive tumor screening for genital lesions is strongly recommended to facilitate early detection and optimize clinical outcomes.

In our study, the most common biopsied skin disease was EMPD. EMPD is a rare cutaneous malignancy with metastatic potential, predominantly affecting elderly individuals in apocrine-rich regions such as the genital, perianal, and axillary areas. This dermatologic entity may either precede or coexist with underlying visceral malignancies (4). Clinically, EMPD can mimic common inflammatory and infectious dermatoses, leading to delays in diagnosis and potentially resulting in metastasis and a poor prognosis. In our study, EMPD was characterized by late onset, long disease duration, and a predilection for the scrotum. A panel of immunohistochemical staining, including AE1/AE3, CK7, CEA, EMA, S100, and periodic acid-Schiff staining, is useful for diagnosing EMPD (5). Additionally, other skin tumors can also occur in the male genitalia area. Mazzoni et al. reviewed a cohort of 1525 patients with genital premalignant and malignant diseases in Australia, providing further context to the range of conditions that can affect this region (6). In our study, premalignant or malignant lesions were identified in 5% of cases (74 cases), with penile intraepithelial neoplasia (57%), SCC (15%), and BP (12%) being the most common. These lesions exhibited distinct anatomical distributions: penile intraepithelial neoplasia and BP were found on the penile shaft, SCC on the glans, and BCC on the scrotum. The benign tumors in our cohort included seborrheic keratosis, angiokeratoma, dermatofibroma, acrochordon (soft fibroma), epidermolytic acanthoma, solitary reticular histiocytoma, keloid, and melanocytic nevus. Malignant neoplasms identified included Bowen’s disease, SCC, BCC, verrucous carcinoma, and cutaneous B-cell lymphoma. Histopathological examination is the golden standard for distinguishing these diseases. Our findings suggested that cutaneous neoplasms in the external genital region may develop either as primary lesions or as secondary manifestations of systemic malignancies.

In our research, the relatively low incidence of classic STIs can be attributed to two key factors. Firstly, these infections are typically diagnosed through serological tests, viral cultures, or nucleic acid amplification tests rather than histopathological examination. Secondly, biopsy is generally unnecessary for their clinical confirmation unless atypical presentations warrant histological evaluation. Therefore, our current study design cannot fully capture the true epidemiological profile of STIs diagnosed by standard laboratory methods. Some atypical manifestations of syphilis, such as Follmann balanitis, phagedenic chancres or multiple chancres, may be misdiagnosed or missed in the study if they were not clinically suspected and therefore not biopsied (7, 8). Among infectious etiologies in our cohort, the most prevalent diagnoses were BP, warts, syphilis, HSV infection, scabies and molluscum contagiosum. BP, a genital dysplasia induced by HPV infection, is characterized by multiple asymptomatic, well-demarcated, red-brown to violaceous papules with a flat, smooth, or verrucous surface. These lesions typically arise on the glans penis and foreskin in male patients and may coalesce into larger plaques (9). In our research, BP was found to have the highest incidence rate among adults aged 18–60 years. Despite its prevalence, we observed a 42% discordance rate between initial clinical impressions and final histopathological diagnoses, primarily due to its morphological similarity to other conditions such as condyloma acuminatum, viral warts, lichen planus, and seborrheic keratosis. Pathologically, BP needs to be differentiated from Bowen disease and erythroplasia of Queyrat. Although the transformation of BP into invasive SCC is rare, occurring in less than 1% of patients, it is still recommended to perform HPV subtyping tests to screen for potential carcinogenic HPV infections. Additionally, concurrent physical examinations and cytologic screening for sexual partners are recommended to ensure comprehensive management and prevention of disease progression (10).

In our study, inflammatory dermatoses were the most common disease category, accounting for 191 out of 572 cases. Therefore, these conditions require particular clinical attention due to their potential for chronicity, functional impairment, and significant impact on quality of life. The predominant entities in our cohort included LS, LP, balanitis, and psoriasis. LS, also referred to balanitis xerotica obliterans, is a chronic inflammatory disorder that affects both genital and extragenital mucocutaneous tissues. In our research, it primarily affected the glans penis and prepuce. LS can present with hypopigmented lesions, erosions, or atrophy, and is characterized by debilitating symptoms such as pruritus, pain, dysuria, urinary stream obstruction, dyspareunia, and sexual dysfunction (11). Importantly, LS is associated with an increased risk of developing squamous cell carcinoma of penis, highlighting the need for vigilant monitoring and management of this condition (12). Early identification of LS is crucial for alleviating symptoms, preventing disease progression, and reducing the risk of malignant transformation. In contrast, psoriasis often presents as well-demarcated, erythematous plaques, with genital involvement affecting up to 63% of patients (13). However, in 2%–5% of patients, genital involvement may be the only manifestation of cutaneous psoriasis (14). This isolated presentation, particularly when it affects the glans penis and coronal sulcus, frequently exhibit atypical clinical features that complicate diagnosis. We observed a 53% discordance rate between initial clinical impressions of genital psoriasis and histopathological diagnoses. In such challenging cases, the presence of extragenital psoriatic features, such as nail pitting, arthropathy or classic skin lesions, often provides valuable diagnostic clues.

In addition, our study observed several rare conditions, including epidermolytic acanthoma, solitary reticular histiocytoma, verruciform xanthoma, and porokeratosis. However, the limited sample size of these cases presents certain limitations in our research findings. Despite these constraints, the identification of these rare conditions contributes to a broader understanding of dermatological disease spectrum.

In our study, 198 patients (34.6%) showed discrepancies between clinical and histological diagnoses. This inconsistency may be due to the clinical similarities among various male genital dermatoses. To address this challenge, dermoscopy and reflectance confocal microscopy (RCM), as non-invasive diagnostic tools, can be used to evaluate both melanocytic and non-melanocytic skin lesions, as well as a range of inflammatory and infectious skin diseases. Our analysis revealed a 57.5% diagnostic concordance rate (42/73) between dermoscopic features and histopathological confirmation, underscoring the clinical utility of dermoscopy in characterizing prototypical genital lesions such as LS, SCC, LP and melanocytic nevus. However, diagnostic discrepancies persisted in cases with atypical presentations or non-specific dermoscopic patterns (e.g., inflammatory dermatoses and early malignant transformations), necessitating multimodal integration of clinical-pathological correlation. Notably, the limited adoption of concurrent dermoscopic-histopathological evaluation (16.9%, 73/433 cases) highlights systemic gaps in real-world diagnostic workflows. By identifying characteristic patterns, dermoscopy and RCM significantly improve diagnostic accuracy and help to minimize the need for unnecessary invasive procedures. Extensive literature has documented the dermoscopic and RCM features of common genital diseases, providing valuable references for their diagnosis and differential diagnosis, thereby enhancing the precision of dermatological assessments (9, 15). We have reviewed and summarized the most common male external genital skin disorders in our study in the Table 5 (16–23). Given the unique nature of the anatomical area, some scholars have suggested using polarized, non-contact dermoscopy to prevent the spread of infectious diseases. Additionally, videodermatoscope can be used to avoid close contact between the examiner’s head and the patient’s genitals (16).

**TABLE 5:** Dermoscopic, reflectance confocal microscopic and histopathological characteristics of the most common six diseases in the study.

Diagnosis	Characteristics of dermoscopy	Characteristics of reflectance confocal microscopy	Characteristics of histopathology
Extramammary Paget’s disease	Milky-red background with polymorphic vascular patterns (dotted/glomerular vessels) and shiny white streaks (strawberry-field pattern) surface scales, ulcers. Psoriasiform scaling and erosive foci overlying structureless white zones.	Dark holes and atypical honeycomb pattern in the epidermis. Low-signal spots located sporadically in the high signal lines of the basal layer. Glandular nests in the dermoepidermal junction and superficial dermis.	Large intraepidermal Paget cells with abundant basophilic/amphophilic cytoplasm and vesicular nuclei containing prominent nucleoli lying singly or in clusters in the epidermis.
Bowenoid papulosis	Pigmented papillomatous surface with slate-gray globules and glomerular or hair pin-shaped vessels. Focal brown-gray dots.	Acanthosis, alteration of the honeycomb pattern, with the presence of multiple irregular bright cells in the dermal papillae.	Full-thickness epidermal dysplasia with crowded pleomorphic keratinocytes, suprabasal mitoses, and bowenoid nuclear atypia. Focal melanic pigmentation in the epidermis, dilated blood vessels in the papillary dermis.
Lichen sclerosus	Porcelain-white structureless areas with cigarette-paper wrinkling and arborizing telangiectasia. Follicular keratosis (yellow comedone-like plugs) and purpuric dots, globules or blotches.	Atypical honeycomb pattern, absent or obscured edged papillae, and scattered small bright cells in the dermis.	Atrophic epidermis with basal vacuolization, homogenized eosinophilic collagen in the upper dermis, and lichenoid lymphocytic infiltrate.
Lichen planus	Violaceous background with linear pearly-whitish structures (Wickham striae), radial dotted/linear vessels, and gray-blue dots.	Large polygonal cells containing a luminous grainy cytoplasm in the granular layer, a dense infiltrate of plump bright cells arranged in sheet-like structures in the dermal-epidermal junction.	Wedge-shaped hypergranulosis, liquefaction and degeneration of basal cells, dense lymphocytes infiltration in a band-like manner in the dermoepidermal junction.
Genital warts	Papillomatous or finger-like structures with dotted/loop or hairpin-like blood vessels surrounded by white edges and red/black dots.	A broadened epidermis with bright epidermal cells and hypervascularization.	Papillomatous acanthosis with hyperkeratosis, parakeratosis, obvious koilocytes, dilation of superficial dermal vessels and superficial dilated vessels surrounded by chronic inflammation.
Squamous cell carcinoma	Irregular, white/yellow structureless areas and irregular twisted/hairpin-like/spiral/dot-spherical vessels. Focal bright white streaks and hemorrhagic crusts.	Disordered epidermal stratification, atypical honeycomb or a disarranged pattern; irregularly shaped clusters of hyper-reflective cells; dilated, round blood vessels.	Disordered epidermal architecture with invasive nests of dysplastic keratinocytes showing nuclear pleomorphism and abundant mitoses. Different depths of invasion and infiltration can be seen in different stages.

## Conclusion

In conclusion, our research presents a comprehensive single-center retrospective study encompassing 572 histopathologically confirmed cases of male external genital skin lesions. The primary objective of this study was to enhance diagnostic accuracy and minimize misdiagnosis by thoroughly examining the demographic, clinical, and histopathological features of these conditions.

## Data Availability

The original contributions presented in this study are included in this article/Supplementary material, further inquiries can be directed to the corresponding author.

## Supplementary material

Supplementary Figure 1 Dermoscopic features of common male external genital skin diseases. **(a)** Extramammary Paget disease: Erythematous background with dotted and glomerular vessels interspersed with milky-white structureless areas with scales. **(b)** Bowenoid papulosis: Hyperkeratotic surface displaying slate-gray pigmentation in annular/granular patterns. **(c)** Lichen sclerosus: Porcelain-white structureless areas with “cigarette-paper” wrinkling, surrounded by arborizing telangiectasia and polymorphic vessels. **(d)** Lichen planus: Violaceous background, bright white linear structures, peripheral brown-gray peppering, and short linear vessels. **(e)** Genital warts: Papillomatous projections with central dotted/looped vessels encircled by whitish halos, small hemorrhagic spots, and a few white scales. **(f)** Squamous cell carcinoma: Erythematous background with disorganized irregular dotted/globular vessels, amidst focal bright white streaks, hemorrhagic crusts, and slightly scales.

Supplementary Figure 2 Histopathological features of common male external genital skin diseases (haematoxylin-eosin stain, × 10). **(a)** Extramammary Paget disease: Intraepidermal proliferation of multiple large pale-staining Paget cells with abundant cytoplasm, arranged singly or in nests. **(b)** Bowenoid papulosis: Full-thickness epidermal dysplasia with multiple pleomorphic keratinocytes, atypical mitoses, and dilated papillary dermal capillaries. **(c)** Lichen sclerosus: Epidermal atrophy with basal vacuolization, hyalinization accompanied melanin incontinence in the papillary dermis, and perivascular lymphocytic infiltration below. **(d)** Lichen planus: Wedge-shaped hypergranulosis, basal layer liquefactive degeneration, and band-like lymphohistiocytic infiltrate obscuring the dermoepidermal junction. **(e)** Genital warts: Acanthosis with papillomatosis, hyperkeratosis, and koilocytic atypia, dilation of superficial dermal vessels and chronic inflammatory infiltration in the upper dermis. **(f)** Squamous cell carcinoma *in situ*: Full-thickness epidermal atypia with disordered maturation, dyskeratotic cells, and mitotic figures above the basal layer.
