# Supplementary figures and images for: Male external genital skin disorders: a retrospective analysis from a tertiary hospital in China
Source: Front Med (Lausanne). 2025 Dec 3;12:1630632. doi: 10.3389/fmed.2025.1630632 (PMC12708296; doi:10.3389/fmed.2025.1630632)

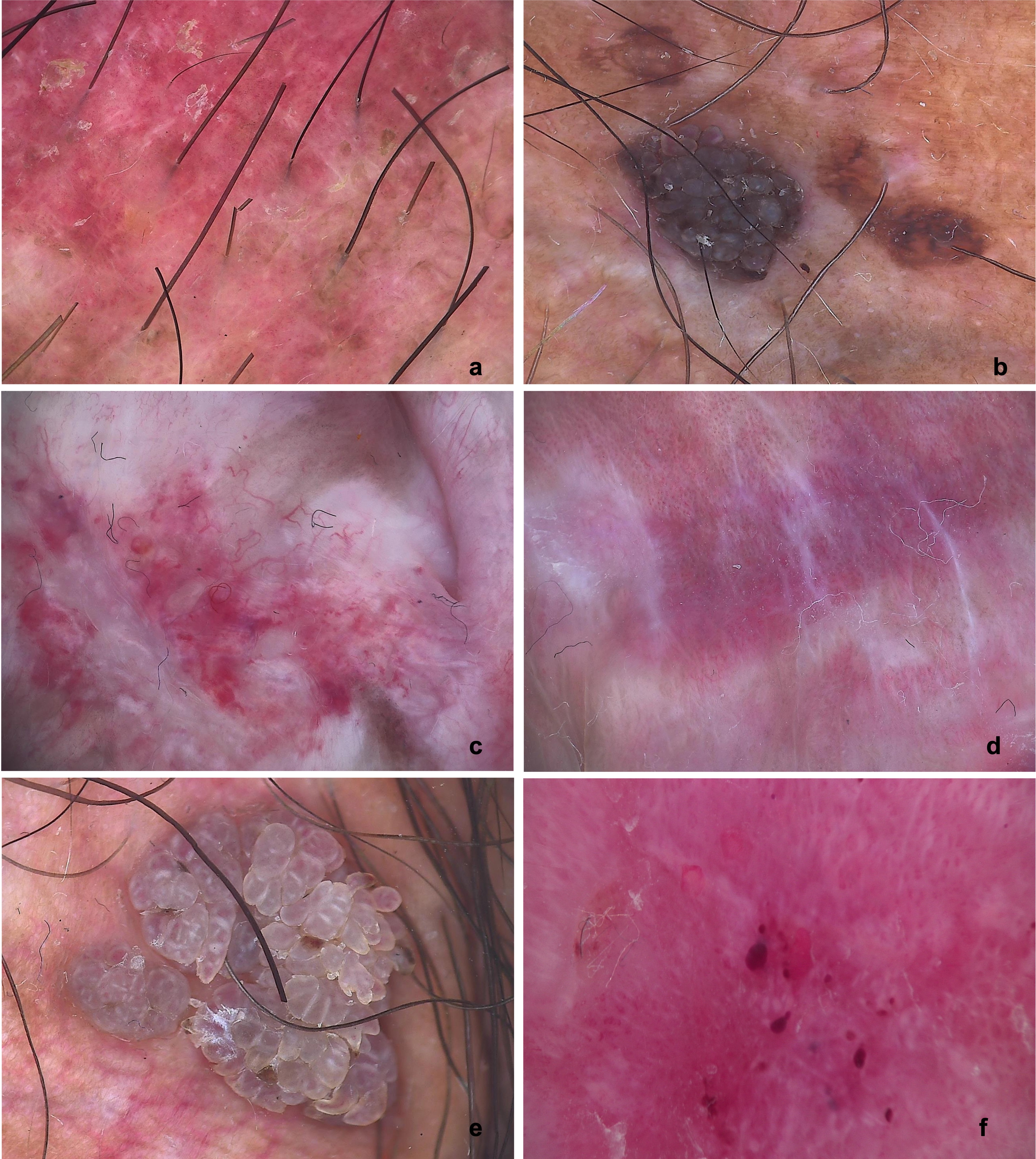

Supplement: Supplementary Figure 1 — Dermoscopic features of common male external genital skin diseases. (a) Extramammary Paget disease: Erythematous background with dotted and glomerular vessels interspersed with milky-white structureless areas with scales. (b) Bowenoid papulosis: Hyperkeratotic surface displaying slate-gray pigmentation in annular/granular patterns. (c) Lichen sclerosus: Porcelain-white structureless areas with “cigarette-paper” wrinkling, surrounded by arborizing telangiectasia and polymorphic vessels. (d) Lichen planus: Violaceous background, bright white linear structures, peripheral brown-gray peppering, and short linear vessels. (e) Genital warts: Papillomatous projections with central dotted/looped vessels encircled by whitish halos, small hemorrhagic spots, and a few white scales. (f) Squamous cell carcinoma: Erythematous background with disorganized irregular dotted/globular vessels, amidst focal bright white streaks, hemorrhagic crusts, and slightly scales. [file Image_1.jpeg]

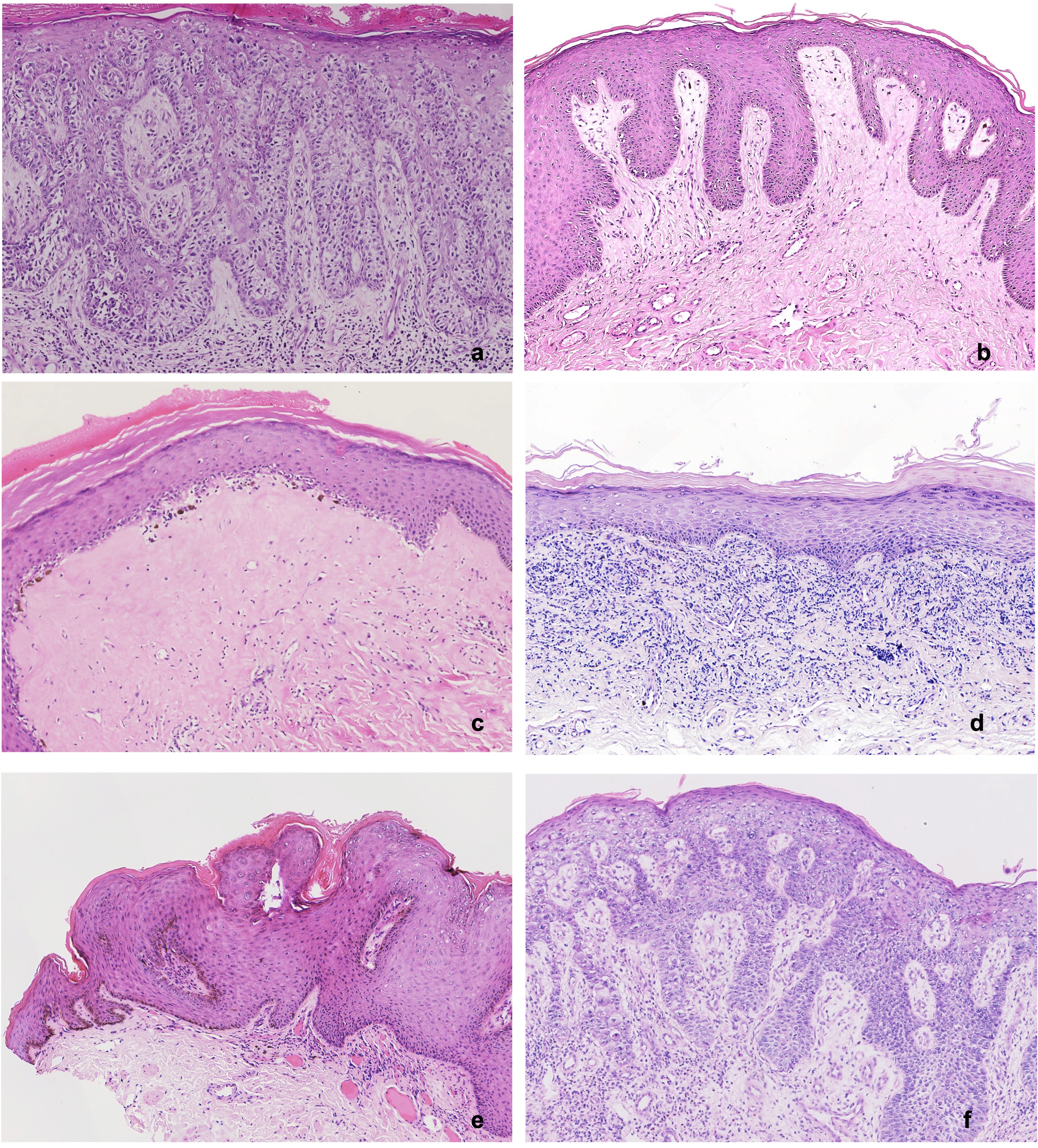

Supplement: Supplementary Figure 2 — Histopathological features of common male external genital skin diseases (haematoxylin-eosin stain, × 10). (a) Extramammary Paget disease: Intraepidermal proliferation of multiple large pale-staining Paget cells with abundant cytoplasm, arranged singly or in nests. (b) Bowenoid papulosis: Full-thickness epidermal dysplasia with multiple pleomorphic keratinocytes, atypical mitoses, and dilated papillary dermal capillaries. (c) Lichen sclerosus: Epidermal atrophy with basal vacuolization, hyalinization accompanied melanin incontinence in the papillary dermis, and perivascular lymphocytic infiltration below. (d) Lichen planus: Wedge-shaped hypergranulosis, basal layer liquefactive degeneration, and band-like lymphohistiocytic infiltrate obscuring the dermoepidermal junction. (e) Genital warts: Acanthosis with papillomatosis, hyperkeratosis, and koilocytic atypia, dilation of superficial dermal vessels and chronic inflammatory infiltration in the upper dermis. (f) Squamous cell carcinoma in situ: Full-thickness epidermal atypia with disordered maturation, dyskeratotic cells, and mitotic figures above the basal layer. [file Image_2.jpeg]
